# [^68^Ga]Ga-DOTA-TOC: The First FDA-Approved ^68^Ga-Radiopharmaceutical for PET Imaging

**DOI:** 10.3390/ph13030038

**Published:** 2020-03-03

**Authors:** Ute Hennrich, Martina Benešová

**Affiliations:** 1 Division of Radiology, German Cancer Research Center (DKFZ), Im Neuenheimer Feld 280, 69120 Heidelberg, Germany; 2 Molecular Biology of Systemic Radiotherapy Group, German Cancer Research Center (DKFZ), Im Neuenheimer Feld 280, 69120 Heidelberg, Germany; m.benesova@dkfz.de

**Keywords:** [^68^Ga]Ga-DOTA-TOC, positron emission tomography (PET), somatostatin receptor (SSTR), neuroendocrine tumors (NETs), theranostics

## Abstract

In the United States, [^68^Ga]Ga-DOTA-TOC has been approved by the Food and Drug Administration (FDA) in 2019 as the first ^68^Ga-radiopharmaceutical for imaging of somatostatin receptor (SSTR) positive gastroenteropancreatic neuroendocrine tumors while employing positron emission tomography (PET). In Europe (Austria, Germany, France), [^68^Ga]Ga-DOTA-TOC was already approved back in 2016. This radiopharmaceutical combines the radionuclide ^68^Ga with the somatostatin analogue DOTA-TOC for specific imaging of tumor cells expressing SSTRs. Such a targeting approach can also be used for therapy planning in the case of both localized as well as disseminated disease and potentially for the evaluation of treatment response.

## 1. Introduction

On 21 August 2019, [^68^Ga]Ga-DOTA-TOC was approved by the Food and Drug Administration (FDA) [1] for positron emission tomography (PET) imaging of somatostatin receptor (SSTR)-positive gastroenteropancreatic neuroendocrine tumors. Holder of the marketing authorization is the UIHC–PET Imaging Center (University of Iowa Health Care (UIHC)), in Iowa, USA. The ready-to-use ^68^Ga-labeled peptide was already approved in some European countries (Austria, Germany, and France) in 2016 (IASOtoc^®^, IASON GmbH, Graz, Austria) [2] and in 2018 (TOCscan^®^, ITM AG, München, Germany) [3]. Also in Europe, a kit preparation for ^68^Ga-labeling of DOTA-TOC (SomaKit TOC^®^, AAA, a Novartis company, Saint-Genis-Pouilly, France) was approved by the European Medicines Agency (EMA) on 8 December 2016 [4]. Use of this kit along with an authorized ^68^Ge/^68^Ga-generator enables on-site preparation of [^68^Ga]Ga-DOTA-TOC even in small facilities. Similar to Europe, in the U.S. a kit preparation for ^68^Ga-labeling of DOTA-TATE (NETSPOT^TM^, AAA, a Novartis Company, Saint-Genis-Pouilly, France) was approved by the FDA on 1 June 2016 [5]. These kits allow decentralized tracer production and therefore enable the application of the radiotracer to patients who do not live in the vicinity of a centralized production site. 

**Figure 1:**
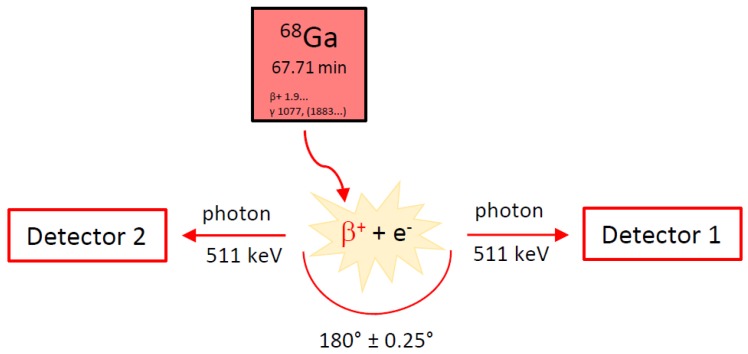
Principle of positron emission tomography (PET). β^+^: positron, e^−^: electron from surrounding tissue.

PET is a commonly used imaging technique in nuclear medicine. It allows a non-invasive and quantitative imaging of cellular and molecular events in patients [6], giving functional information in contrast to morphological information obtained from conventional imaging techniques like computed tomography (CT) or magnetic resonance imaging (MRI). For this imaging method so-called PET tracers, which are biological molecules or sometimes artificial building blocks for specific targets labeled with positron emitters (e.g., gallium-68, fluorine-18), are intravenously injected into the patient. These radioactive nuclides decay by positron emission. After a certain reach in tissue (depending on the nature of both the radionuclide and the tissue), the positron will interact with an electron and this interaction will result in an annihilation event. During this process two annihilation photons with an energy of 511 keV each are emitted in an angle of 180° ± 0.25° (see Figure 1). PET systems use multiple detectors connected by a coincidence circuit that are distributed in opposite directions and encircle the patient. Only if two detectors opposite to each other detect a signal within a very short coincidence time (nanoseconds), a positron decay is registered. This imaging technique is ideal to monitor molecular events in early disease stages as well as treatment response [6]. An optimal PET tracer should be easy to produce and to radioactively label. Other important properties of such a PET tracer are demonstrated by a rapid uptake in targeted tissue (high specificity), a rapid clearance from non-targeted tissue, a high stability in vivo, and by an absence of host-immune response. These properties enable the imaging of the patient shortly after radiotracer administration and minimize the dose to the patient, who is free to go home right after the examination is finished [7]. For proper diagnostics, the radiotracer should demonstrate high target-to-background ratios, whereas the absolute uptake into the targeted tissue does not necessarily have to be high [7].

The PET tracer [^68^Ga]Ga-DOTA-TOC is not only suitable for imaging, but also for a so-called theranostic approach, which has gained high importance in recent years. Radiotheranostics thus represents the epitome of personalized (precision) nuclear medicine [8]. The initial step of such a radiotheranostic approach is to employ a targeting molecule labeled with a diagnostic radionuclide (e.g., gallium-68, technetium-99m), which is used for quantitative imaging of a tumor-related biomarker, either with PET or single photon emission computed tomography (SPECT). Positive accumulation of the radiolabeled targeting molecule in tumor lesions is followed by the administration of the same or an analogical targeting molecule, labeled with a therapeutic radionuclide that delivers a tumoricidal radiation dose to the malignant tissue. In other words, molecular imaging helps to distinguish the patients who will benefit from the treatment from those who will most probably not. Those patients already show insufficient uptake of the tracer in the PET scan. Such a pair of theranostic tracers is represented, among others, by the diagnostic [^68^Ga]Ga-DOTA-TOC and its therapeutic partner [^177^Lu]Lu-DOTA-TATE (DOTA-(D-Phe^1^,Tyr^3^)-octreotate, Lutathera^®^ (AAA, France)). [^177^Lu]Lu-DOTA-TATE was approved by the EMA and the FDA in 2017 and 2018, respectively, as the first radiopharmaceutical for peptide receptor radionuclide therapy [9,10,11]. In the case of this theranostic pair, one cannot speak of a true pair of identical theranostic radiopharmaceuticals since the imaging tracer uses the SSTR binding peptide DOTA-TOC, while the therapeutic peptide uses DOTA-TATE instead. Both peptides show high binding affinity to human SSTR2 (for comparison: IC_50_ (Ga-DOTA-TOC) = 2.5 ± 0.5 nM and IC_50_ (Ga-DOTA-TATE) = 0.2 ± 0.04 nM) [12]. While Ga-DOTA-TATE shows only affinity to SSTR2, Ga-DOTA-TOC also exhibits some affinity to SSTR5 [12]. Despite their different binding affinities, a head-to-head comparison of [^68^Ga]Ga-DOTA-TOC and [^68^Ga]Ga-DOTA-TATE showed no clinically significant difference between the two tracers [13]. Therefore, in this case the use of different substances as a theranostic pair is possible. Currently, the enhanced implementation of theranostics in nuclear medicine is generally well appreciated.

Neuroendocrine tumors (NETs) demonstrate a group of tumors originating from neuroendocrine cells that are distributed throughout the human body. Approximately two-thirds of NETs derive from the gastrointestinal system and represent the group of gastroenteropancreatic tumors (GEP-NETs) [14]. Most of the NETs demonstrate high expression levels of SSTRs, offering the possibility of molecular imaging and peptide receptor radionuclide therapy using radiolabeled somatostatin analogs [15]. Five subtypes of SSTRs were described in the literature: SSTR1 to SSTR5, out of these SSTR2, 3 and 5 are known to differ from SSTR1 and 4 when considering their amino acid homology and pharmacological profile [16]. SSTR2 and SSTR5 are the most commonly expressed subtypes in NETs, despite a considerable variability in the expression of SSTR subtypes in different kinds of tumors [17]. NETs were previously considered to be a rare disease, but their incidence and prevalence have significantly increased over the last decade. The development of radiolabeled somatostatin ligands has been a very long scientific journey, starting with the first description of somatostatin 45 years ago [18]. Until now, multiple diagnostic as well as therapeutic somatostatin ligands have been developed and some of them were summarized in a review by Kwekkeboom et al. [19]. The first radiolabeled SST analog used for imaging was [^123^I, Tyr^3^]octreotide in 1989 [20]. Since then, a lot of different SST tracers have been developed for imaging with SPECT as well as PET. The advancement of these tracers was a milestone for the diagnosis of NETs. The much higher sensitivity of SPECT for the detection of NETs and metastases compared to conventional imaging techniques like CT, MRI, and ultrasound was demonstrated in numerous studies [21]. The most commonly used tracer for SPECT is [^111^In]In-DTPA-octreotide (OctreoScan^®^, Curium^TM^ (UK); first marketing authorization Mallinckrodt Medical B.V. (Netherlands): 1994 in the U.S. and 1995 in Europe). However, OctreoScan^®^ has several drawbacks, such as moderate binding affinity for SSTR2 and insufficient coordination of therapeutic beta emitters like yttrium-90 and lutetium-177 [22]. The replacement of DTPA with the macrocyclic chelator DOTA resulted in DOTA-(D-Phe^1^,Tyr^3^)-octreotide (DOTA-(Tyr^3^)-octreotide, DOTA-TOC), DOTA-(D-Phe^1^,Tyr^3^,Thr^8^)-octreotate (DOTA-TATE), and DOTA-(1-Nal^3^)-octreotide (DOTA-NOC) [23]. Even though all three peptides labelled with ^68^Ga show very good results in PET imaging [24], DOTA-TOC and DOTA-TATE have progressed further. These two prominent SST analogs are extensively used in clinical nuclear medicine for both diagnosis and therapy. The most promising PET tracers are represented by [^68^Ga]Ga-DOTA-TOC and [^68^Ga]Ga-DOTA-TATE. One of their advantages—in comparison to the SPECT tracers—is a higher affinity for SSTR2, which is most commonly expressed by NETs [19]. Another advantage lies in the superior image quality of PET scans vs. SPECT scans, which leads to an increased sensitivity for tumor detection [19]. The peptide DOTA-TOC was developed by the group of Maecke and labelled with indium-111 for imaging as well as yttrium-90 for peptide receptor radiotherapy (PRRT), showing promising results [25,26]. It was the first time a peptide was complexed with yttrium-90 [25]. The chemistry and biological evaluation of the peptide DOTA-TOC labelled with gallium-67, indium-111, and yttrium-90 was described by Heppeler et al. in 1999 and [^67^Ga]Ga-DOTA-TOC showed superior properties in comparison to OctreoScan [27]. First imaging of patients with [^68^Ga]Ga-DOTA-TOC was reported by Hofmann et al. in 2001 [28]. This study in eight patients demonstrated a lower kidney accumulation and higher tumor-to-background ratio compared to [^111^In]In-DTPA-octreotide. Since then, [^68^Ga]Ga-DOTA-TOC has been widely used in Europe [29]. To support [^68^Ga]Ga-DOTA-TOC approval by the FDA, a meta-analysis of the tracer efficacy was conducted by UIHC–PET Imaging Center [29]. This study designated [^68^Ga]Ga-DOTA-TOC as an excellent tracer for the assessment of patients with known NETs and for the planning of patients´ management. 

**Figure 2:**
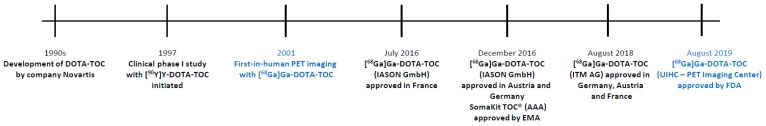
DOTA-TOC timeline. The first use for in-human imaging with [^68^Ga]Ga-DOTA-TOC and its FDA approval are highlighted in blue.

Moreover, DOTA-TOC was selected as a model substance for first-in-human PET/CT imaging with cyclotron-produced ^44^Sc ([^44^Sc]Sc-DOTA-TOC) and radiolanthanide ^152^Tb ([^152^Tb]Tb-DOTA-TOC), both performed in Germany [30,31]. However, the broader use of these two exotic radionuclides in the clinical practice is still in question. Their superiority compared to clinically-established diagnostic radionuclides within a bigger pool of patients is yet to be proven, just as is their future availability in high enough quantities. Finally, therapeutic variants including alpha particle-emitting [^213^Bi]Bi-DOTA-TOC and [^225^Ac]Ac-DOTA-TOC were also evaluated in clinical scenarios [32,33]. Despite limited data available, the treatment with low-LET β^−^-particle-emitters (e.g., ^177^Lu) shows only average success in clinical trials and, thus, more effort is put into the evaluation of high-LET α-particle-emitters (^213^Bi, ^225^Ac). The higher efficiency of α-particle-emitters to destroy cancer tissue represents a clear advantage, especially for patients with highly aggressive or resistant cancer disease [34,35]. Figure 2 gives a short timeline for the SSTR agonist DOTA-TOC, starting from its development in the 1990s [36], over its use as a therapeutic ([^90^Y]Y-DOTA-TOC) [36], to the FDA approval of diagnostic [^68^Ga]Ga-DOTA-TOC in 2019.

## 2. Chemical Overview

### 2.1. Names and Structure

**Figure 3:**
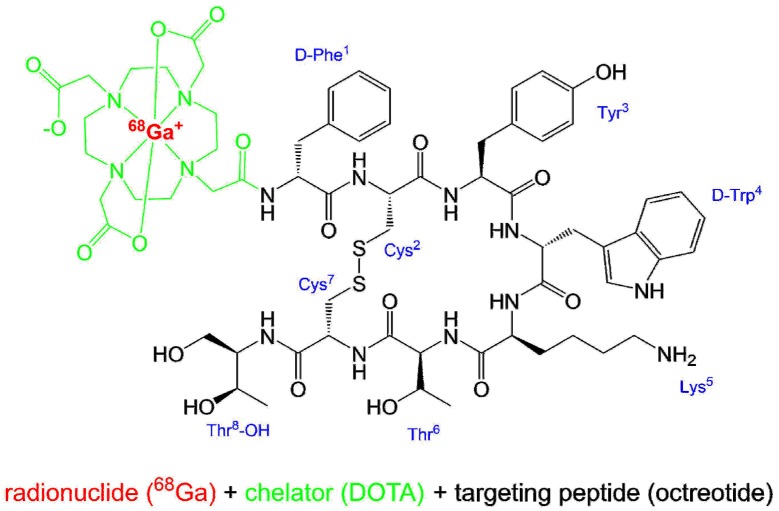
Structure of [^68^Ga]Ga-DOTA-TOC.

Ready to use [^68^Ga]Ga-DOTA-TOC (Figure 3) is commercially available by UIHC–PET Imaging Center (Iowa, USA). In some European countries, [^68^Ga]Ga-DOTA-TOC is commercially available by IASON GmbH (IASOtoc^®^, Austria) and ITM AG (TOCscan^®^, Germany). In addition, the peptide DOTA-TOC as a kit preparation for labeling with gallium-68 (SomaKit TOC^®^, AAA, France) is available in Europe. Other names for [^68^Ga]Ga-DOTA-TOC are ^68^Ga-Edotreotide or [^68^Ga]Ga-DOTA-(D-Phe^1^,Tyr^3^)-octreotide, and its amino acid sequence is D-Phe-cyclo(Cys-Tyr-D-Trp-Lys-Thr-Cys)Thr(ol). The IUPAC name of [^68^Ga]Ga-DOTA-TOC is following:

[^68^Ga]gallium-N-[(4,7,10-tricarboxymethyl-1,4,7,10-tetraazacyclododec-1-yl)acetyl]-D-phenylalanyl-L-cysteinyl-L-tyrosyl-D-tryptophyl-L-lysyl-L-threonyl-N-[(1R,2R)-2-hydroxy-1-(hydroxymethyl)propyl]-L-cysteinamide cyclic(2-7)disulfide.

The radionuclide gallium-68 is complexed by the bifunctional chelator DOTA (1,4,7,10-tetraazacyclododecane-1,4,7,10-tetraacetic acid), which acts as a hexadentate chelator with octahedral geometry, coordinating gallium-68 to four nitrogen atoms as well as two carboxylic groups [37]. The [^68^Ga]Ga-DOTA complex is bound to the somatostatin affine peptide (D-Phe^1^,Tyr^3^)-octreotide. Figure 3 shows the structure of [^68^Ga]Ga-DOTA-TOC in simplified drawing for geometry [37] and complexation [38] of [^68^Ga]Ga^3+^ in the DOTA moiety. 

### 2.2. Gallium-68

The radionuclide gallium-68 (^68^Ga) decays with a half-life of 67.7 min through positron emission to the stable isotope zinc-68 (^68^Zn) [39]. The positron yield is 89.1% and while the maximal β^+^ energy is 1899 keV, the average is 836 keV [40]. This results in a mean positron range of 1.05 mm in soft tissue, before the annihilation of a positron with an electron takes place [40].

A huge advantage of gallium-68 is its availability by use of a ^68^Ge/^68^Ga-generator, compared to the use of common cyclotron-produced PET nuclides (e.g., fluorine-18 and carbon-11). Thus, also small facilities without a cyclotron on site are able to produce PET tracers. These generators are commercially available. For the production of ^68^Ga-labeled radiopharmaceuticals under GMP (Good Manufacturing Practice) conditions, authorized generators are on the market. Generators GalliaPharm^®^ (Eckert & Ziegler, Berlin, Germany) or Galli Ad^®^ (IRE ELit, Fleurus, Belgium) are available in Europe. In the U.S., FDA-approved versions are GalliaPharm^®^ (Eckert & Ziegler, Berlin, Germany) or Galli Eo^®^ (distributed by Cardinal Health, Dublin, OH, USA). With a half-life of 271 d, the parent nuclide germanium-68 (^68^Ge) continuously decays via electron capture (EC) to the daughter nuclide ^68^Ga, which can be eluted using diluted hydrochloric acid multiple times a day (regeneration needs approximately three half-lives of ^68^Ga) [41]. One generator can be used for about one year when a reduction of the obtainable activity to less than 1/2 resulting in a decrease of patient doses is acceptable. All in all, ^68^Ga has a great potential as it may compete with the most utilized cyclotron-produced radioisotope fluorine-18 (^18^F). However, the β+ emission energy of ^68^Ga is higher than that of ^18^F, which can potentially lead to lower spatial resolution. Moreover, despite the fact that ^68^Ge/^68^Ga-generators provide good flexibility, the limited amount of activity loaded on the generator, the necessary intervals between elutions and their efficiency, possible ^68^Ge-breakthrough and, finally, a very high demand on authorized ^68^Ge/^68^Ga-generators resulting in long waiting times clearly limit their utilization and also increase their price (up to about USD 100,000). Based on this situation, another alternative, the large scale cyclotron-based production of ^68^Ga, is currently under development [42]. 

### 2.3. Manufacturing and Quality Criteria

[^68^Ga]Ga-DOTA-TOC is produced in a GMP-compliant automated radiosynthesis by reacting n.c.a. (no-carrier-added) [^68^Ga]GaCl_3_ with DOTA-TOC under suitable conditions. A detailed description of the automated radiosynthesis of [^68^Ga]Ga-DOTA-TOC including quality control is given in a protocol for a theranostic clinical phase II trial conducted by UIHC–PET Imaging Center [43]: After elution from the ^68^Ge/^68^Ga-generator, the eluate is purified using an ion-exchange column and transferred to the reaction vial containing DOTA-TOC. The reaction mixture is heated to 95 °C for 7 min. Following the reaction, the product is purified by solid phase extraction (C-18 cartridge), formulated, and sterile filtered. The radiochemical purity and other parameters of the final sterile product solution are controlled. Only if all quality criteria are fulfilled will the product be released for medical application. The product specifications of [^68^Ga]Ga-DOTA-TOC produced by UIHC–PET Imaging Center are in accordance with the monograph “Gallium (^68^Ga) Edotreotide Injection” (2482) in the *European Pharmacopeia* [44]. The finished product manufactured by UIHC–PET Imaging Center is dispensed into a multiple dose vial containing 259–2072 MBq [^68^Ga]Ga-DOTA-TOC [45] in aqueous solution (10% EtOH v/v in sodium chloride (9 mg/mL), 14 mL) with an activity concentration of 18.5–148 MBq/mL at calibration date and time [46]. The buffered injection solution is sterile, pyrogen free, clear, colorless, and has a pH value of 4–8. The product has a shelf-life of 3 h from the date and time of calibration, which is due to radioactive decay and not instability of the compound (radiochemical purity ~99% after 3 h [45]). Considering the half-life of ^68^Ga, a far-reaching distribution of the radiotracer is not feasible.

In Europe it is also possible to manufacture [^68^Ga]Ga-DOTA-TOC by using the kit preparation SomaKit TOC^®^ (AAA, France), which allows the labeling of the peptide with ^68^Ga (authorized ^68^Ge/^68^Ga-generator) in-house. It is not obligatory to do this procedure in a clean room environment, only a controlled environment is necessary because both the kit and the eluate from the generator are sterile. It is also unnecessary to purify or sterile filter the product solution. For quality control, only minimal testing (appearance, pH value and determination of radiochemical purity by thin layer chromatography) is required and the product quality does not have to reach all the parameters defined in the monograph “Gallium (^68^Ga) Edotreotide Injection” (2482) in the *European Pharmacopeia* [44]. 

## 3. Medicinal and Pharmaceutical Overview

### 3.1. Clinical Indication

[^68^Ga]Ga-DOTA-TOC is a radioactive diagnostic tracer for the localization of somatostatin receptor positive neuroendocrine tumors in adult and pediatric patients using PET [46].

### 3.2. Application

The following application recommendations are taken from the prescribing information of [^68^Ga]Ga-DOTA-TOC [46]. [^68^Ga]Ga-DOTA-TOC is administered as an intravenous injection with a recommended amount of radioactivity of 148 MBq (range 111–185 MBq) in adults and of 1.59 MBq/kg (range 11.1–111 MBq) for pediatric patients. Since the radiotracer binds to the same somatostatin receptors as somatostatin analogs, patients who are taking short-acting somatostatin analogs have to discontinue their use 24 h before imaging. Patients who are taking long-acting analogs should be imaged just prior to the next dosing. Patients do not have to be fasting before imaging, but are instructed to drink water to ensure adequate hydration, as well as to continue drinking water following the administration of the radiotracer in order to reduce radiation exposure by frequent voiding. Image acquisition can start at 60 min post injection (p.i.) (range 55–90 min) including the whole body (from skull vertex to mid-thigh).

### 3.3. Pharmacology and Pharmacokinetics

The SSTR subtype affinity profile of a number of somatostatin analogs was investigated by Reubi et al. [12]. DOTA-TOC exhibits high affinity for human SSTR2 (IC_50_ of 14 ± 2.6 nM) with much lower binding affinity for all other human SSTRs. Complexation of this peptide with gallium resulted in an even higher affinity for SSTR2 (IC_50_ of 2.5 ± 0.5 nM). This high affinity for SSTR2 leads to a binding of [^68^Ga]Ga-DOTA-TOC to cells with an upregulation of SSTR2 including malignant cells. Depending on the presence and density of SSTR in the tissue, different amounts of the radioactive tracer are taken up, resulting in variable intensities of the signals in the PET scan. Normal tissues with high physiological uptake of [^68^Ga]Ga-DOTA-TOC include spleen, kidneys, liver, pituitary gland, thyroid gland, and adrenals. High uptake is also seen in the pancreas uncinated process [47]. In most GEP-NETs SSTR2 is upregulated and a higher uptake of the radiotracer compared to normal background can be observed. However, if the density of somatostatin receptors is not sufficient, the lesions cannot be visualized by [^68^Ga]Ga-DOTA-TOC [47]. An increased uptake of [^68^Ga]Ga-DOTA-TOC is not specific for GEP-NETs and, therefore, the evaluation of disease-specific uptake is warranted [46,48]. As [^68^Ga]Ga-DOTA-TOC is an agonist for SSTR, the tracer is internalized upon binding to the receptor [48].

Owing to the nanomolar concentration of the peptide (<50 µg per injection [43] ≙ 3.6 µg/mL, 2.4 nmol/mL) in the sterile injection solution, no clinically relevant pharmacodynamic effects are expected [47].

The pharmacokinetic properties of [^68^Ga]Ga-DOTA-TOC are described, inter alia (i.a.) in the product monograph for SomaKit TOC^®^ [47]. After intravenous injection of the radiotracer rapid clearance from blood following a bi-exponential elimination of the activity (half-lives: 2.0 ± 0.3 min and 48 ± 7 min) can be observed. As mentioned above, the highest uptake of [^68^Ga]Ga-DOTA-TOC is seen in spleen, followed by the kidneys, and approximately 50 min p.i. the tracer accumulation plateaus in all organs. Normal tissue uptake was shown to be age- and gender-independent. Excretion of the tracer occurs via the kidneys as the intact compound with approximately 16% of the radioactivity eliminated in the urine within 2–4 h p.i. No radioactive metabolites could be detected in the serum within 4 h after injection. Since the physical half-life of ^68^Ga (67.7 min) is considerably lower than the elimination rate of the tracer, the biological half-life of the tracer will have little impact on the effective half-life of [^68^Ga]Ga-DOTA-TOC. Effective half-lives of the tracer in liver and kidney are about 70 min and 75 min, respectively [48]. 

Radiation dosimetry of [^68^Ga]Ga-DOTA-TOC was evaluated in a study by Sandström et al. [49]. Organs with the highest radiation absorbed doses are the urinary bladder wall and the spleen. Table 1 summarizes the estimated radiation absorbed doses per injected activity of [^68^Ga]Ga-DOTA-TOC in selected organs.

The typical radiation doses resulting from an injection of 148 MBq of [^68^Ga]Ga-DOTA-TOC to an adult weighing 75 kg are about 18 mSv (urinary bladder wall), 16 mSv (spleen), and 12 mSv (kidneys and adrenals) [46].

## 4. Perspective

The radiolabeling of DOTA-peptides like DOTA-TOC or DOTA-TATE with gallium-68 is a straightforward one-step synthesis. In contrast to other widely used PET nuclides like cyclotron-produced fluorine-18, gallium-68 is available by use of a ^68^Ge/^68^Ga-generator. This also enables small facilities—without access to a cyclotron—the in-house production of their radiotracers. Furthermore, the use of an approved labeling kit and an authorized generator eliminates the need of a dedicated clean room, as a controlled environment may be sufficient. However, it is important to note that for the preparation of a ^68^Ga-tracer, intended for medical use, an authorized ^68^Ge/^68^Ga-generator is mandatory. With the development and introduction of the [^68^Ga]Ga-PSMA-11 radiotracer [50,51,52] used for prostate cancer imaging, the demand for ^68^Ge/^68^Ga-generators increased considerably. This resulted in long waiting times for a generator that is also quite expensive. Another point to consider is the relatively short half-life of gallium-68 and the typical amount of radioactivity that can be eluted from a generator (1.2–1.8 GBq for a fresh generator). This leads to a situation in which only a few patients can be examined with one batch of ^68^Ga-tracer. These potential drawbacks of ^68^Ga-tracers can be avoided when nuclides like cyclotron-produced fluorine-18 (half-life 110 min) as well as suitable ^18^F-labeled tracers are available. But a huge challenge for radiolabeling with fluorine-18 is the radiochemistry, which often includes multi-step syntheses with relatively harsh reaction conditions unsuitable for the labeling of peptides as well as purifications by HPLC. For an easy automation of the synthesis, which is a prerequisite for GMP-compliance, it is necessary to have as few reaction steps as possible. There have been a lot of developments towards ^18^F-labeled somatostatin tracers [53], but for most of them the radiosynthesis is more complicated than a straightforward metal-complexation. Some of these tracers are currently in early clinical trials, e.g., [^18^F]FET-βAG-TOCA [54], which shows promising imaging results, but its complicated production is a drawback. Another tracer, [^18^F]AlF-NOTA-octreotide, which was first developed by Laverman et al. [55] and shows promising imaging results [56,57], may overcome the problems of a complicated radiosynthesis. For this tracer, an automated two-step GMP-compliant synthesis was recently published [58]. The future will show if [^18^F]AlF-NOTA-octreotide is a potential ^18^F-labeled alternative for the imaging of SSTR as is the case for [^68^Ga]Ga-PSMA-11 and [^18^F]PSMA-1007 [59,60]—which can be produced in a one-step GMP-compliant radiosynthesis [61]–for imaging of prostate cancer.

## 5. Conclusions

Approximately three years after the approval in some European countries, the ready-to-use ^68^Ga-labeled peptide [^68^Ga]Ga-DOTA-TOC was approved by the FDA in 2019. Combining the radionuclide ^68^Ga with the somatostatin analog DOTA-TOC, non-invasive and specific imaging of somatostatin receptors in gastroenteropancreatic neuroendocrine tumors and metastases can be accomplished by PET imaging. [^68^Ga]Ga-DOTA-TOC appears to be an excellent tracer for the assessment of patients with known NETs and is useful for planning of patient management within a theranostic approach. Considering the half-life of ^68^Ga (67.7 min), a wide distribution of [^68^Ga]Ga-DOTA-TOC is not feasible. In Europe, the use of an approved kit preparation (SomaKit TOC^®^), together with an authorized ^68^Ge/^68^Ga-generator, represents an option for clinical facilities not in reach of an authorized production site. Thus, the decentralized preparation of [^68^Ga]Ga-DOTA-TOC becomes also viable in small facilities. 

## Figures and Tables

**Table 1:** Estimated radiation absorbed doses for [^68^Ga]Ga-DOTA-TOC in selected organs [49].

Organ	Absorbed Dose/mGy/MBq (n = 9)	
	*Mean*	*SD*
Urinary bladder wall	0.119	0.058
Spleen	0.108	0.065
Kidney	0.082	0.020
Adrenal gland	0.077	0.028
Liver	0.041	0.014
Red Marrow	0.016	0.003
Gallbladder wall	0.015	0.001
Lungs	0.007	0.001
Total Body	0.014	0.002
**Effective Dose**/mSv/MBq	0.021	0.003
